# View dependencies in the visual recognition of social interactions

**DOI:** 10.3389/fpsyg.2013.00752

**Published:** 2013-10-21

**Authors:** Stephan de la Rosa, Sarah Mieskes, Heinrich H. Bülthoff, Cristóbal Curio

**Affiliations:** Department for Perception, Cognition and Action, Max Planck Institute for Biological CyberneticsTübingen, Germany

**Keywords:** visual recognition, view dependent, social interactions, visual cues, action observation

## Abstract

Recognizing social interactions, e.g., two people shaking hands, is important for obtaining information about other people and the surrounding social environment. Despite the visual complexity of social interactions, humans have often little difficulties to visually recognize social interactions. What is the visual representation of social interactions and the bodily visual cues that promote this remarkable human ability? Viewpoint dependent representations are considered to be at the heart of the visual recognition of many visual stimuli including objects (Bülthoff and Edelman, 1992), and biological motion patterns (Verfaillie, 1993). Here we addressed the question whether complex social actions acted out between pairs of people, e.g., hugging, are also represented in a similar manner. To this end, we created 3-D models from motion captured actions acted out by two people, e.g., hugging. These 3-D models allowed to present the same action from different viewpoints. Participants' task was to discriminate a target action from distractor actions using a one-interval-forced-choice (1IFC) task. We measured participants' recognition performance in terms of reaction times (RT) and d-prime (d'). For each tested action we found one view that led to superior recognition performance compared to other views. This finding demonstrates view-dependent effects of visual recognition, which are in line with the idea of a view-dependent representation of social interactions. Subsequently, we examined the degree to which velocities of joints are able to predict the recognition performance of social interactions in order to determine candidate visual cues underlying the recognition of social interactions. We found that the velocities of the arms, both feet, and hips correlated with recognition performance.

## Introduction

Humans are social beings who daily physically interact with other humans, e.g., when greeting a friend with a hug. We refer to these non-verbal physical interpersonal interactions such as shaking hands, kissing, or fist fighting as social interactions. The visual recognition of physical social interactions is important for humans to successfully navigate through their social and physical environment, for example, when avoiding a street in which people are fighting. Shedding light onto the visual processes underlying visual social interaction recognition helps understanding how humans achieve this feat.

Relatively little is known about the visual processes underlying the visual recognition of social interactions. Previous research has mainly focused on elucidating the mechanisms underlying the recognition of individual actions. For example, research on biological motion highlighted the importance of dynamic information in the recognition of individual actions, e.g., walking (Blake and Shiffrar, 2007), and a large body of neuroscientific work has examined the contributions of the motor system to action recognition of individual actions (Kozlowski and Cutting, 1978; Prinz, 1997; Gallese and Goldman, 1998; Keysers et al., 2010—see Jacob and Jeannerod, 2005; Mahon and Caramazza, 2008; Hickok, 2009 for a debate on this issue). However, the visual recognition of social interactions has received scant attention (Neri et al., 2006).

There is strong evidence that the underlying representation of visual recognition is view-dependent (for alternative views on this topic see e.g., Biederman, 1987). View dependencies in visual recognition are taken as evidence for the underlying visual representation being tuned to certain views and thereby highlight an important organizational principle of visual recognition. Previous research has shown view dependencies in the recognition of static objects (Bülthoff and Edelman, 1992; Tarr and Buelthoff, 1995), and faces (Hill et al., 1997). A special kind of view dependency, namely orientation sensitivity, has been demonstrated for the visual recognition of static body postures (Reed et al., 2003). Overall, view dependencies in the recognition of static visual patterns are frequently observed and indicate that the underlying visual representation is view-dependent.

Actions are inherently dynamic resulting in a change of body posture over time. This biological motion information is considered to be critical for action recognition. Point light displays are an elegant way to examine the ability of the visual system to recognize biological motion (Johansson, 1973) as these displays consist mainly of motion but little structural information. Previous research using point light displays demonstrated that biological motion informs the observer about the executed action (Dittrich, 1993) and about socially relevant information. For example, humans are able to readily recognize the gender (Kozlowski and Cutting, 1977, 1978; Pollick et al., 2005), emotions (Atkinson et al., 2004), and the identity (Loula et al., 2005; Jokisch et al., 2006) of point light walkers.

View dependencies have also been reported for the recognition of dynamic biological motion patterns of walkers (Verfaillie, 1993; Troje et al., 2005). For example, identification of biological motion patterns was found to be better for the trained view than for novel views (Troje et al., 2005). In the light of recent suggestions that the motor systems contributes to action understanding (Kozlowski and Cutting, 1978; Prinz, 1997; Gallese and Goldman, 1998; Keysers et al., 2010—although this view is not undebated: Jacob and Jeannerod, 2005; Mahon and Caramazza, 2008; Hickok, 2009), recent evidence from a physiological study can be interpreted in favor for view-dependencies in visual action recognition. Here, Caggiano et al. (2011) reported that in monkeys motor-visual neurons, which are considered to be important for action understanding (Gallese et al., 2004), exhibit view-dependent responses when processing object-directed actions. However, view dependencies in the visual recognition of dynamic social actions occurring between pairs of people, such as two person shaking hands, has not been investigated yet.

Here we examined viewpoint dependencies in the recognition of complex social actions, in particular social interactions. We motion captured interactions (high five; handshake; hug) acted out by pairs of participants and created 3-D models of these interactions. In the actual experiment, participants saw these interactions one at a time and had to report whether the shown interaction matched a predefined interaction (1IFC task). We manipulated the type of interaction (hug, high-five, or handshake) and the viewpoint [behind, side, top, 45° (angled) view] from which participants saw the interaction. Moreover, because previous research has shown that visual recognition is very rapid (Thorpe et al., 1996; Furl et al., 2007; de la Rosa et al., 2011), we probed the visual recognition of interactions at different presentation times. We recorded participants' accuracy (as measured by d-prime) and reaction time to identify a predefined social interaction. We reasoned that if visual recognition of social interaction is view-dependent, then recognition performance should vary with the viewpoint.

If visual recognition of social interactions is view-dependent, then low level visual cues (e.g., velocity of bodily joints projected onto the viewing plane), which change over different views, might be able to explain the visual recognition of social interactions. To answer the question, which visual cues might support the recognition performance of social interactions, we analyzed the physical body motion patterns to determine candidate visual cues that participants might have used for the recognition of social interactions. Specifically, we calculated the velocity of each joint (e.g., left elbow) after it had been projected on the viewing plane. We used these velocities and also the correlations between opponent joints (e.g., left and right elbow) velocities as predictors for the recognition performance observed in the experiment. The latter velocities had been calculated since movement of opponent limbs is considered to be critical for the action recognition of point light actors (Casile and Giese, 2005). In addition, we correlated the joint velocities between corresponding joints of the two actors (e.g., the velocity of Person's A left elbow with the velocity of Person's B left elbow). The purpose of these correlations was to capture the temporal synchronization between actors, which influences social interaction recognition (Neri et al., 2006). We then used the velocities of individual joints, opponent joint correlations, and the correlation between corresponding joints as predictors for the recognition performance obtained in experiment. We expected that joints that correlate highly with the recognition performance are candidate visual cues that participants might have used for the visual recognition of social interactions.

## Methods

### Participants

Ten right-handed participants (mean age = 26.4; *sd* = 6.6) recruited from the local community in Tübingen participated in the experiment. All participants gave their written informed consent prior to the experiment. All participants had normal or corrected-to-normal vision. The experiment was conducted in accordance with the Declaration of Helsinki.

### Stimuli and apparatus

The stimuli were motion captured actions (handshake, hug, high five) acted out by 17 different pairs of actors using MVN suits (Xsens Technologies B.V., Eschede, Netherlands) (recording sampling rate: 120 Hz). Actors were facing each other in an empty room and always started out from the same resting position, which was a standing pose at a predefined spatial position. A sound served as a start signal for actors to act out one of the three actions as instructed by the experimenter. After the acting, actors went back into their resting position. Each pair of actor acted out each action at least five times.

**Figure 1:**
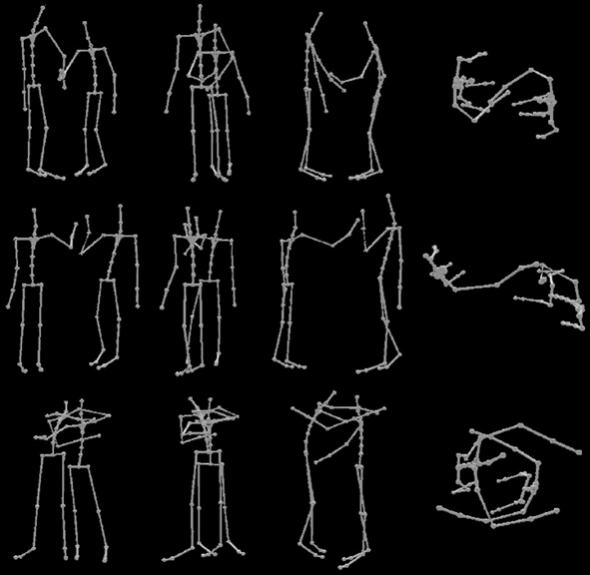
**Example stimuli for each viewpoint and action condition showing the last frame from an action movie used in the experiment**. Different actions are shown across rows (handshake, high five, hug) and different viewpoints of the action are shown across columns (angled, behind, side, top).

The motion capture data was post-processed in Blender and Matlab: The motion capture clips were chopped in such a way that they started displaying 40 ms of resting position and stopped 40 ms after the apex of the interaction had been reached (e.g., the moment when the actors first touched each other). The motion capture was animated using a stick figure by wrapping an orange cylinder around each limb and an orange symmetrical sphere on the joints (see Figure 1). In the end, 5 different 3D models for each of the three interactions (handshake, hug, high five) were used. During the experiment each model was shown 144 times across all experimental conditions. We used the custom written software based on Matlab and the Psychophysics toolbox3 (Brainard, 1997; Pelli, 1997; Kleiner et al., 2007) to display the stimuli in the experiment on a Dell LCD screen using a Dell PC. The Horde3D engine was used within the Psychophysics toolbox to display the stick figure animations from different viewpoints.

### Procedure

Participants sat in front of the computer screen and orally received the following information about the experiment from the experimenter. The experiment consisted of three experimental blocks which were run consecutively on the same day. An experimental block started by presenting a white word in the center of the black computer screen. This word indicated the target interaction (hug, high five, or handshake) that participants had to look out for during the experimental block. After pressing the space bar the screen turned black and the experiment started by presenting the experimental trials. An experimental trial consisted of the presentation of an interaction (two stick figures on a black screen) followed by a two second answer interval, which consisted of a black screen. If participants think they saw the target interaction, they pressed the “>” key and “z” otherwise (1IFC). Participant could give their answer at any time during the presentation. The reaction time was measured from the beginning of the presentation. The pressing of an answer key resulted in the immediate skipping forward to the end of the current trial. Trials were separated by a 500 ms inter-stimulus-interval consisting of a black screen. An experimental block consisted of 144 experimental trials. Participants were instructed to answer as accurately and fast as possible.

Each experimental block tested the recognition of one of the three interactions. Every participant had to recognize every interaction. The testing order of interactions (and hence experimental blocks) was randomized across participants. Every experimental block tested all possible combinations of the two factors of interest: four viewpoints (profile, half profile, behind, top; see Figure 1) and five presentation times (0.4, 0.6, 0.8, 1.0, 1.61 s). Each combination of viewpoint and presentation time was tested 36 times within an experimental block. 18 of these 36 trials were target trials and 18 were non-target trials. The 18 non-target trials consisted of a counterbalanced presentation of the two non-target interactions. Hence an experimental block consisted of 4^*^5^*^36 = 720 trials. The trials were presented in random order. Participants completed three of these experimental blocks (each aiming for a different action and randomized order across participants) for a total of 2160 trials. The experiment lasted around 2 h.

The factors viewpoint, action, presentation time were completely crossed within subject factors. The dependent variables were reaction time (measured in s) and accuracy (as measured by d′).

## Results

We removed trials in which participants did not give an answer or where responses were faster than 200 ms (5% of the total trials). These latter responses were considered uninformed guesses.

### Reaction times

**Figure 2:**
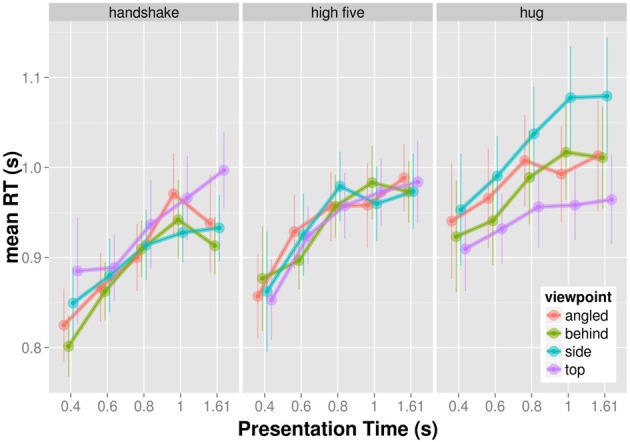
**Mean reaction times as a function of presentation time shown for each action and viewpoint separately**.

The mean reaction times (RT) for trials, in which participants correctly identified the target, are shown in Figure 2. Reaction time increases with increasing presentation time for all actions and viewpoints. The effect of viewpoint on RT seems to vary across actions. We examined the observed RT patterns by means of a three-factorial completely crossed within subjects ANOVA. Viewpoint, action, and presentation time served as within subject factors and mean RT as a dependent variable. We report Greenhouse-Geisser corrected *p*-values in order to counteract observed violations of sphericity. The main effect of presentation time, *F*_(4, 36)_ = 18.863, *p* > 0.001, and action, *F*_(2, 18)_ = 9.618, *p* = 0.004, were significant. The main effect of viewpoint was non-signficant, *F*_(3, 27)_ = 1.322, *p* = 0.290. The interaction between action and presentation time, *F*_(8, 72)_ = 0.716, *p* = 0.564, and the interaction between presentation time and viewpoint, *F*_(12, 108)_ = 1.192, *p* = 0.331, were non-significant. However, the interaction between action and viewpoint was significant, *F*_(6, 54)_ = 4.786, *p* = 0.005. The three-way interaction between viewpoint, action, and presentation time was non-significant, *F*_(24, 216)_ = 1.109, *p* = 0.369. The significant interaction between action and viewpoint indicates that viewpoint influences visual recognition for some actions more than for others.

**Figure 3:**
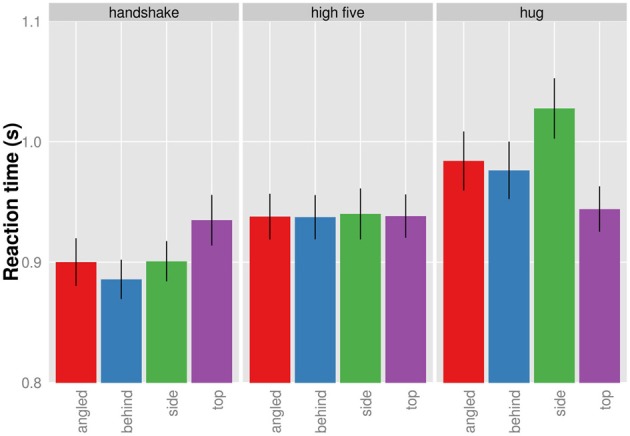
**Mean reaction times given for each action and viewpoint separately (collapsed across presentation duration)**. Bars indicated one standard error from the mean.

Figure 3 shows the significant interaction between presentation and action. Clearly, the effect of viewpoint on action was different across the three actions. While there seems to be little influence of viewpoint on RT for the recognition of high five actions, the RT to hug and handshake actions seem to depend on the viewpoint. Specifically, the behind view resulted in the shortest RT for handshakes and the top view resulted in the shortest RT for the hugs. We conducted all pairwise comparisons of viewpoints for each action separately using the Holm correction. For handshake and high five actions, none of the viewpoint comparisons produced a significant result (*p* > 0.05). The only significant difference was found for the recognition of hug actions when viewed from the top compared to when viewed the profile view, *t*_(9)_ = 3.77; *p*_Holm corrected_ = 0.026. The top view allowed faster recognition. Overall the analysis of RT shows a significant effect of viewpoint on RT for the recognition of hug actions.

### D-prime

**Figure 4:**
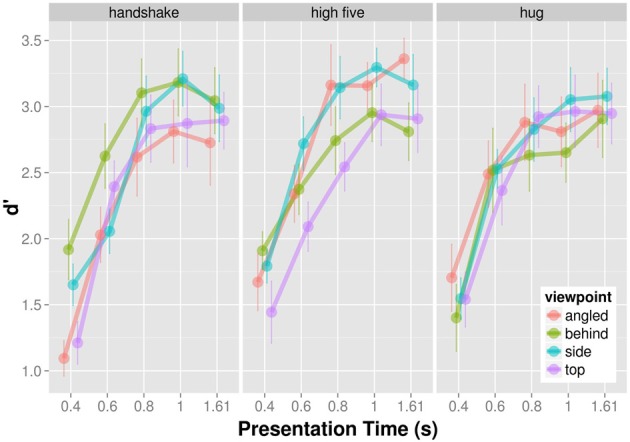
**Mean d′ as a function of presentation time shown for each action and viewpoint separately**.

D′ provides a measure for participants' ability to discriminate the target from non-targets with higher *d*′ values indicating better discrimination performance. The mean d′ increases as a function of presentation time for each viewpoint and action (Figure 4) indicating that participants were better able to tell the target from non-targets with increasing presentation time. The d′-presentation time curves seem to differ for different viewpoints of the same action.

We analyzed the effect of presentation time, viewpoint, and action on d′ in a completely crossed within subject ANOVA. Because we observed deviations from sphericity for some effects, we report Greenhouse-Geisser corrected *p*-values where appropriate. The main effect of presentation time, *F*_(4, 36)_ = 60.52, *p* < 0.001, and angle was significant, *F*_(3, 27)_ = 3.59, *p* = 0.027. The main effect of action was non-significant, *F*_(2, 18)_ = 0.433, *p* = 0.655. The interaction between action and presentation time, *F*_(8, 72)_ = 1.279, *p* = 0.268, and the interaction between viewpoint and presentation time, *F*_(12, 108)_ = 1.111; *p* = 0.367, was non-significant. The interaction between viewpoint and action was significant, *F*_(6, 54)_ = 6.670; *p* < 0.001. The three way interaction between viewpoint, action, and presentation time was non-significant, *F*_(24, 216)_ = 0.956; *p* = 0.468. Overall, the ANOVA suggests that discrimination of target from non-targets dependent on the particular combination of action and viewpoint.

**Figure 5:**
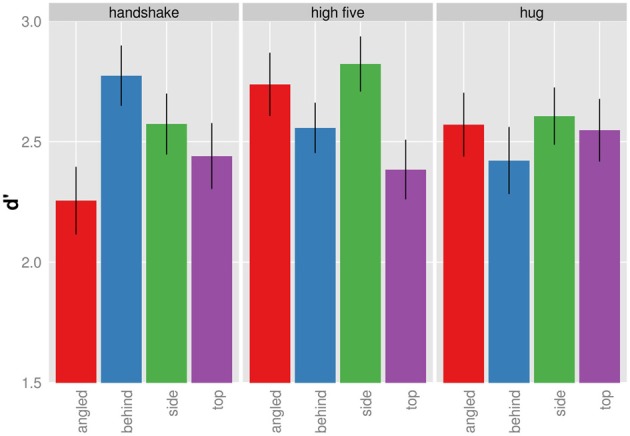
**Mean d′ given for each action and viewpoint separately (collapsed across presentation duration)**. Bars indicated one standard error from the mean.

Figure 5 shows the significant interaction between action and viewpoint. The modulation of d′ with viewpoint is stronger for the recognition handshakes and high-fives than for the recognition of hugs. To examine the effect of viewpoint on d′ in more detail we conducted all pairwise comparison of the four viewpoint d′ for each action separately using the Holm correction. As for handshakes, we only found the following comparisons to be significant. D′ of top views were significantly higher than of angled views, *t*_(9)_ = 3.396; *p* = 0.040, and d′ of behind views were significantly higher than of angled views, *t*_(9)_ = 5.149; *p* = 0.004. High-fives when seen from the side view were associated with a significantly higher d′ than when seen from the top, *t*_(9)_ = 4.513; *p* = 0.009. None of the other viewpoint d′ comparisons for high five actions were significant (*p* > 0.05). Finally we did not find any differences with respect to d′ differences across viewpoints for hugging actions (*p* > 0.05). In sum the d′ analysis demonstrates viewpoint modulations of d′ for the recognition of handshake and high-five actions.

### Correlating physical motion patterns with recognition performance

The experiment suggests that social interaction recognition performance is not the same across all views and therefore, support the hypothesis that not all social interactions are represented in a view invariant fashion. What are candidate visual cues that participants might have used for the visual recognition of social interactions? One reasonable assumption is that visual cues used during the visual recognition of social interactions should correlate with observed recognition performance. Specifically, we used the velocities of bodily joints (e.g., the left elbow) to predict the observed recognition performance of the previous experiment. We used joint velocities within our analysis because biological motion information as provided by joint movements are critical for the recognition of actions (Blakemore and Decety, 2001; Blake and Shiffrar, 2007).

We used linear regression to predict recognition performance from joint velocities. We employed three types of predictors. First, we used the velocity of individual joints (individual joint velocities). Second, we correlated the velocities of joints that occur on both the left and right side of the same body (e.g., left and right elbow) across time because previous research suggest that opponent or antiphase movement of these joints is critical for action recognition (Giese and Poggio, 2003; Casile and Giese, 2005; Thurman and Grossman, 2008). We will refer to this type of correlation as opponent joint correlations. As the third type of predictor served the correlation between the velocities of corresponding joints from the two actors of the same pair (joint velocity correlations). The motivation for the use of this type of predictor stems from previous research demonstrating the importance of temporal synchrony between actions of the interaction partners for the visual recognition of social interactions (Neri et al., 2006). Because we only used symmetric interactions in which both actors were doing very similar movements, one way to measure this interpersonal synchrony is by measuring the velocities of corresponding joints on the two actors across time, e.g., correlate the velocity of the right elbow of Person A with the velocity of the right elbow of Person B.

In the following analysis, we combined RT and d-prime into a single performance measure (RT corrected d prime) to facilitate the interpretation of our results using a univariate regression analysis instead of a multivariate regression analysis.

## Methods

### Body model

For the analysis we used the joints as specified by the bvh body model provided by MVN. This body models consisted of 23 joints (see spheres in Figure 1).

### Data acquisition

We used MATLAB and the Motion Capture Toolbox (Lawrence, 2009) to determine the 3D position of each joint for each action, view, actor, and animation frame. Note that an individual joint can move because the actor is moving as a whole (global body motion) or because the joint itself is moving (local joint movement). Moreover, note that the global position of the body in 3D space of the MVN bvh body model is given by the position of the central hip joint. In order to separate global body motion from the local limb movement, we subtracted the hip 3D space coordinates form all other joint 3D coordinates. As a result, the hips indicate global body position while the remaining joints indicate local joint movement. We then calculated the 2D projections of the 3D joint positions on the viewer's plane. Subsequently, the velocities of each joint in 2D space was calculated as the Euclidean distance of the 2D positions between two successive animation frames. All analysis was carried out on these joint velocities that were obtained for each action, view, actor, and animation frame.

### Data analysis

We used linear regression to predict recognition performance from joint velocities. Because we had two measures of recognition performance in the experiment (d prime and reaction time), we combined the two measures into a single measure for the sake of ease of interpreting univariate regression results: We calculated the *d* prime values adjusted for reaction time by using reaction time as a covariate in the d prime ANOVA model of the results section of the experiment. In particular, we used the inverted reaction time as a covariate to ensure that for both reaction time and d prime, better performance is associated with a larger values. The adjusted mean *d* prime values served as the dependent variable for the regression analysis.

For the individual joint velocity predictors we integrated the velocities for each joint, actor, action, and view across time. Specifically, this integration of velocities was done for the five probed presentation times (0.4, 0.6, 0.8, 1.0, 1.61 s) separately. The opponent joint velocities were simply the correlation of corresponding left and right joints across time calculated for each presentation time separately. As for the joint velocity correlations, we calculated the correlations between velocities of corresponding joints on the two actors of the same pair across time. This correlation was calculated separately for each of the five probed presentation times. The resulting joint velocities and joint velocity correlations were then used as predictors. There were 8 opponent joint velocity predictors, 23 individual joint velocity predictors, and 23 correlated joint velocity predictors.

In the frist step, stepwise multiple regression was used to reduce the initial set of 54 predictors to a smaller set of predictors suitable for recognition performance prediction. We used stepAIC function from the statistical package R with forward and backward elimination using the Akaike information criterion (AIC) as selection criterion. The starting model for the stepwise regression procedure was one that contained only the intercept. Stepwise regression returned a model with 33 predictors explaining 98.68% of the variance in the recognition performance. In a second step, we simplified this model using all subset regression. The best subset regression returned the best model for all possible model sizes (1–33). A visual inspection of the plot of adjusted-*r*^2^ and the BIC criterion (of the best fitting models) against the model size (not shown here) showed that adjusted-*r*^2^ and BIC changed only slightly with model size (i.e., the function asymptotes) for models containing more than 15 predictors (benefit of a model with 16 parameters compared to one with 15 parameters: *r*^2^: less than 0.75% at a 94% fit; BIC less than −4.29 at a BIC value of −105). Hence, we chose the best fitting model with 15 predictors as our final model.

Relative importance of the predictors of the final model were determined by calculating the average *r*^2^ across all predictor orderings (Grömping, 2006). Relative importance measures the average amount of variance in the recognition scores explained by predictor.

## Results

**Figure 6:**
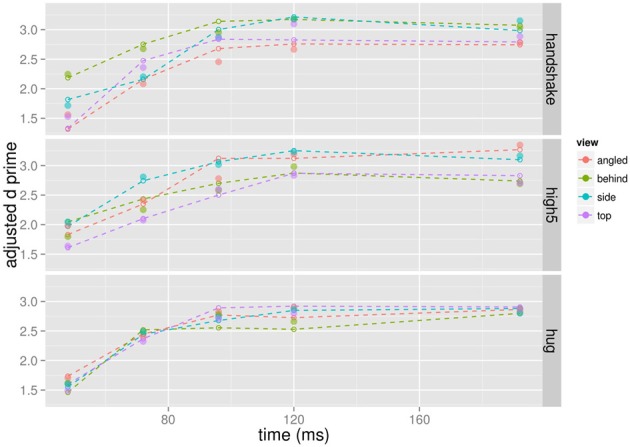
**d′ values adjusted for mean RT of the behavioral experiment (dotted lines and crosses) and the predicted d' performance from the regression model (solid circles)**.

**Figure 7:**
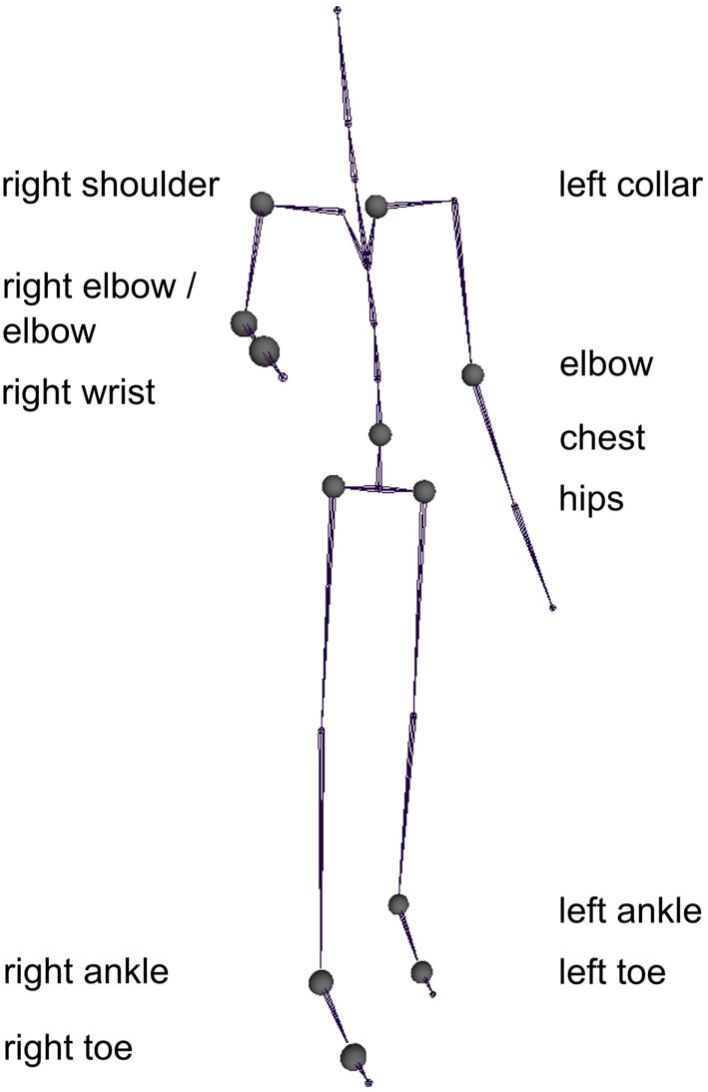
**The joints whose velocity profile was most predictive for the recognition performance as observed in the experiment**.

The final model contained 15 predictors from 11 joints and explained 94.22% of the variance of the *d* prime values (Figure 6). The predictors are listed in Table 1 and the corresponding joints are shown in Figure 7. The model contained predictors referring to individual joint velocities, opponent joint velocities, and joint velocity correlations. These predictive joints were mostly located on the feet, the right arm, and the upper body. The relative importance of each predictor as measured by the average *R*^2^ across different predictor orderings indicated that the individual joint velocities of the right wrist explains most of the variance in the recognition performance. Other important predictors as measured by relative importance were opponent joint movements of hips (i.e., left and right hip) and feet. On average, individual joint velocities and opponent joint correlations explained most of the variance (Table 1) and the correlated joints velocities explained a smaller amount of variance in the recognition performance. Interestingly, the distance between the two persons did not turn out to be a significant predictor of recognition performance as indicated by the lack of the central hip as a predictor in the model. In summary, this analysis demonstrates that low level visual cues (individual joint movements and correlated joint movements) provide one possible explanation for the view-dependent visual recognition of social interactions including the the optimal view of social interactions.

**Table 1:** **Predictors of the final model used to predict recognition performance from joint velocities**.

**Predictor**	**Pr(>|t|)**	**Relative importance (average *r*^2^)**
1. Right wrist	6.97E-014	0.14873565
2. Ankle (opponent joint correlation)	1.64E-006	0.09743588
3. Hip (opponent joint correlation)	9.85E-006	0.09048241
4. Left toe	1.78E-011	0.0824363
5. Right elbow	1.10E-011	0.08204858
6. Toe (opponent joint correlation)	0.000406	0.07884528
7. Right toe	3.75E-008	0.07465615
8. Right ankle	8.14E-006	0.06588832
9. Right shoulder	3.34E-007	0.05590034
10. Chest	0.000177	0.05561856
11. Right ankle (joint velocity correlation)	0.005193	0.02904772
12. Elbow (opponent joint correlation)	2.83E-006	0.02521843
13. Left collar (joint velocity correlation)	0.0007	0.02314838
14. Left toe (joint velocity correlation)	0.000103	0.02093676
15. Right wrist (joint velocity correlation)	2.83E-006	0.01180915

If no predictor type is specified in brackets, predictors refer to individual joint velocities.

**Figure 8:**
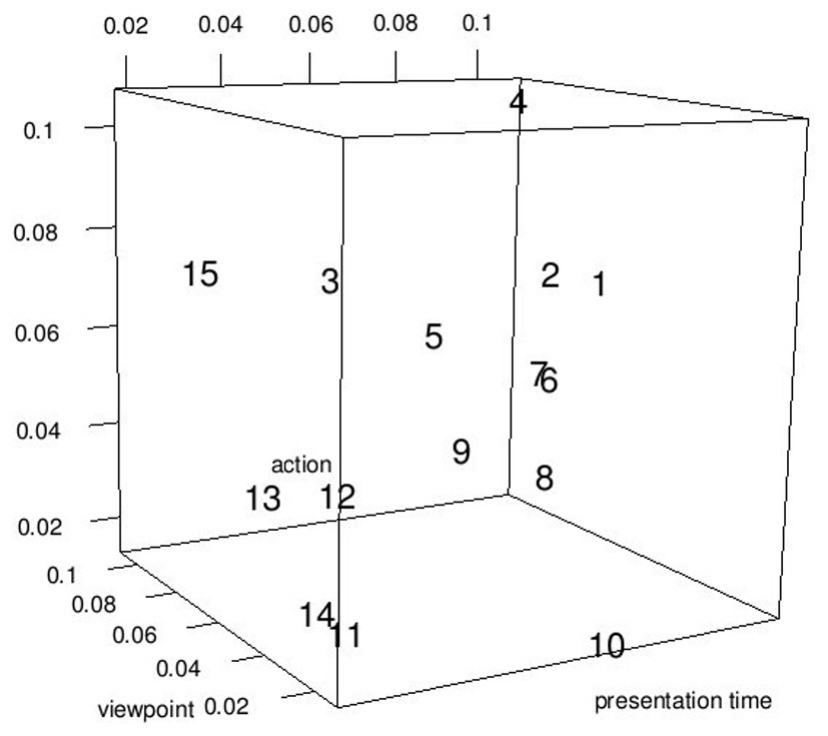
**Relative importance of each predictor (numbers) of Table 1 shown for each experimental factor separately**. The numbers refer to the predictor number in Table 1.

So far we assessed the predictors in terms of their ability to predict the overall recognition performance, i.e., the recognition performance across all experimental conditions. However, it is likely that not all joints are equally informative across all experimental conditions. For example, the hands might be more informative for recognition performance changes across different viewpoints than across different actions. To gain greater insight about which predictors are most predictive for individual experimental factors, we addressed the question to what degree the predictors listed in Table 1 are able to predict performance changes for each experimental factors separately (viewpoint, presentation time, action). For the experimental factor under scrutiny, we regressed recognition performance on the two other experimental factor (not under scrutiny) and obtained the residuals from the fitted model. Note that these residuals have been adjusted for all experimental effects except for the experimental factor in question and therefore, carry mostly information about the experimental factor in question. Using these residuals, we regressed recognition performance on the predictors in Table 1. Finally, we determined the relative importance of each predictor, to determine the predictor is most predictive for the recognition performance changes across the experimental factor in question. The results are shown in Figure 8. The 15 predictors have different predictive power across the three experimental manipulations. The best predictor for performance changes across different viewpoints is the opponent hips correlation. The right wrist is best for predictor for different recognition performances across presentation times. Finally, the end of left foot best predicted changes in recognition performance across different actions. The high predictability of the end of left foot arises from the different walking patterns associated with different actions. For example, actors stepped often only one step forward during a high five and handshake action while taking several steps when conducting a hug action. Moreover, the step was faster during the handshake than during the high five action.

Overall, the results of this analysis suggest that different predictors are associated with different predictive power for different experimental manipulations. Yet, it is noteworthy that the predictor “end of left foot” is overall a good predictor for all three experimental manipulations since it is associated with high relative importance for all three experimental manipulations.

## Discussion

The experiment investigated view dependencies in the visual recognition of hug, handshake, and high-five actions. The experiment showed that RT magnitude depended on the viewpoint for hug actions and that d′ values depended on the viewpoint for high five actions and handshake actions. Hence, the viewpoint had an effect on either RT or d′ for each social interaction. These viewpoint modulations of recognition performance are in line with the idea that not all social interactions are recognized in a view invariant manner.

The analysis of the relationship between joint velocities and recognition performance revealed several candidate movements that offer a possible explanation for view-dependent visual recognition of social interactions based on low level visual cues. In particular, we found joints relating to the right hand, the feet, and to the upper body to be most informative about recognition performance. This result aligns with previous reports about critical sources of visual information for the recognition of biological motion (see below). We also found that the correlation of velocities of opponent limbs are particularly predictive of recognition performance. Although we did not demonstrate a causal relationship between opponent movement and recognition performance in the present study, this finding is in line with the suggestion that anti-phase limb movements are critical for biological motion recognition (Casile and Giese, 2005; Thurman and Grossman, 2008). In addition, we also found that movements between corresponding joints on the two persons (in particular the arm, left collar, and the feet) were predictive of recognition performance as it is expected from studies on the temporal synchrony of interactive movements (Neri et al., 2006). In summary, all three types of velocities are predictive of recognition performance, which is in line with previous reports that opponent movement and the temporal relationships between interaction partners are important. Moreover, the bodily location of the visual cues as suggested by this analysis are in line with previous experimental results showing that the movement of extremities (leg and arms) (Troje and Westhoff, 2006; Thurman and Grossman, 2008) and upper body (Thurman et al., 2010) is diagnostic for the recognition of point light walkers and stick figure walkers.

Additional analysis showed that the predictors are to different degrees indicative of recognition performance changes along the three experimental manipulations (viewpoint, presentation time, and action). For example, the opponent movement of the hips was particularly predictive for performance changes across viewpoints, the end of left foot was most predictive for the performance changes across actions, and the right wrist was indicative of performance changes with changes in the presentation time. Hence not all predictors were equally effective for performance changes associated with the three experimental manipulations.

This physical analysis of the velocity patterns is a first step to understand which visual cues participants relying on during the visual recognition of social interactions. We suggest candidate visual cues that carry information about social interactions and therefore, might be used for the recognition of social interactions. Although these cues are able to explain the view-dependent recognition of social interactions on a theoretical level, additional experiments are required to verify whether participants indeed use these visual cues for the recognition of social interactions.

### Can the observed view-dependencies solely explained by visual learning of local bodily cues?

An alternative explanation of the findings in our study is that participants learned informative local bodily cues, e.g., the movement patterns of the feet, and recognized actions based on these local cues. If participants learned these local cues in a view dependent manner and rely on them during recognition, one would expect recognition performance of social interactions to depend on the viewpoint. However, this type of view-dependent recognition performance would not speak to a view-dependent encoding of social interactions but rather to a view-dependent encoding of local bodily cues.

To asses the plausibility of learning of local bodily cues, we examined the effect of stimulus on recognition performance in the current experiment. First, we reasoned that if participants learned to pick up subtle local differences due to the repeated presentation of the stimuli, we would expect the observed effects to change if the same analysis was conducted on a more limited data set for which the number of stimulus repetitions was much smaller (and hence learning was less pronounced). We analyzed only the first 3 experimental blocks of each participant. For the first 3 experimental blocks, all three interactions were tested for 8 out of the 10 participants (note that interaction presentation order was randomized across participants). The subsequent analysis was, therefore, carried on the 8 participants for which we had data for every interaction. The average repetition rate of stimuli within this limited data set was 15 repetitions per stimulus (compared to 36 repetitions in the full data set). We ran the same analyses on this more limited data set as reported in the results section. The RT analysis on this limited data set showed the same significant effects as the original analysis. Likewise, the d prime analysis exhibited the same pattern of significant effects with the limited data set as it did with the full data set. Hence restricting the analysis to the first three experimental blocks of the data did not change the pattern of the results.

Second, we addressed the issue of learning more directly by conducted a mixed linear model analysis for both d prime and RT data separately. We reasoned that if learning occurred, the performance should improve the more often participants participated in experiments and therefore, the more often they saw the stimuli. The frequency of participants' engagements in the experimental task was measured by the variable experimental block. In both the RT and d-prime analysis, participant was a random effect and experimental block was a fixed effect, whose intercept and slope was allowed to vary in a by-participant manner (i.e., the effect of block was modeled for each participant separately). The results show a non-significant effect of block on both the RT [*t*_(61)_ = −1.68; *p* = 0.09] and the d-prime [*t*_(61)_ = 1.225, *p* = 0.225] analysis suggesting that RT and *d*-prime values did not change significantly across time. It, therefore, seems unlikely that performance largely changed with repeated presentation of the stimuli.

Finally, one could argue that learning is rapid and, therefore, is completed within the first experimental block. We therefore, looked at the change of RT for correct target identifications over trials within the first experimental block. We ran a mixed effects model with trial number as a fixed factor and participant as a random factor. The intercept and slope for trial was fitted in a by-participant fashion to model the performance change over time for each participant separately. The analysis showed a non-significant effect of trial number indicating that RT did not change significantly over time, *t*_(1115)_ = −1.09, *p* = 0.275. We did not calculate the analysis for d prime because the d prime analysis requires averaging of participants' responses across a reasonable number of stimulus repetitions, which was not given for the data of the first block.

Overall, the results of the additional analyses showed little evidence for profound learning effects. We, therefore, think that the observed effects are unlikely due to learning alone and that participants did not rely solely on learned local visual cues during social interaction recognition.

### View-dependent encoding of social interactions or view-dependent encoding of individual actions?

The recognition of individual actions as displayed by point light walkers are known to be view-dependent (Verfaillie, 1993). One could therefore, argue that the view-dependent recognition of social interactions is simply due to the view-dependent visual recognition of the individual actions. Is the view-dependent recognition of social interactions due to view-dependent recognition of individual actions or due to view-dependent recognition of the interactions? There is evidence that the recognition of social interactions is more than the simply the recognition of the constituent individual actions. If the recognition of an interaction were simply the recognition of the constituting individual actions, there should be no interaction between the interaction and the recognition of individual actions. However, this is not the case. Neri et al. (2006), for example, showed that synchronized (meaningful) compared to desynchronized (meaningless) interactions lead to better recognition performance of an individual action constituting the interaction. Because the meaningfulness of an interaction influences the recognition of individual actions, their results suggests that the visual recognition of social interactions is more than merely the recognition of individual actions. In a similar vein, Manera et al. (2011) showed that participants' were better to discriminate a point light actor from noise if this actor was preceded by a communicative compared to a non-communicative agent. Seeing the dyad rather than merely the participating individuals also positively influenced the recognition of emotions within interpersonal dialog. Clarke et al. (2005) showed that the recognition of love and joy was impaired if the interaction partner was not shown. Therefore, perceiving the another person's action as part of a communicative interaction improves recognition performance. Overall, these result provide accumulating evidence that the visual recognition of interactions goes beyond the mere recognition of the individual actions.

In line with these previous results, our analysis of the joint velocity patterns suggests that correlations between velocities of corresponding joints on the two actors are predictive of recognition performance. Hence, joint velocity correlations between the two actors carry social interaction specific information, which could potentially be used by participants in the visual recognition of social interactions.

Despite the ability of these of joint velocity correlations to predict recognition performance, individual joint velocities and opponent joint velocities explain the bulk part of the recognition performance. This suggests that view-dependent recognition of individual actions might also contribute to the view-dependent recognition of social interactions. Taken together, it seems plausible to assume that both view-dependent processing of individual actions as well as view-dependent processing of the interactions contribute to the view-dependent recognition of social interactions. However, a more detailed examination is needed to tease apart the contributions of these two sources to the recognition of social interactions. Our physical analysis provides a starting point for this investigation by suggesting candidate visual cues that correlate with recognition performance.

Motor-visual neural populations are often considered to be critical for the recognition of actions although this view is debated (Jacob and Jeannerod, 2005; Mahon and Caramazza, 2008; Hickok, 2009). If one adopts the former view, the observed view dependent recognition of social interaction recognition align with recent observations in macaque monkeys and humans. Caggiano and colleagues found that the majority (74% of the tested neurons) motor-visual neurons in area F5 exhibit view-dependent response behavior (Caggiano et al., 2011). In humans, a recent study demonstrated view-dependence of visuo-motor cortical areas involved in action observation and action execution (Oosterhof et al., 2012) using fMRI. Our findings also add to the larger growing body of evidence that view-dependent encoding of visual information is an underlying principle for several stimuli including objects (Bülthoff and Edelman, 1992), faces (Troje and Bülthoff, 1996), body postures (Reed et al., 2003), and biological motion (Verfaillie, 1993).

The human ability to read actions from bodily movements is most likely of high relevance in natural social interactions even in the presence of other visual cues conveying social information (e.g., facial expressions). First, facial expressions are indicative of the emotional state of a person. Yet, they do not convey the kind of action that is executed by a person. Hence, the recognition of actions or social interactions requires the recognition of dynamic bodily expressions. Second, the perception of facial expression depends on the immediate situational context, which includes bodily expressions. For example, the emotion that observers associated with a facial expression depended on the body posture and other object context that was presented along with the facial expression (Aviezer et al., 2008). Hence, situational context interacts with facial expression recognition. It seems plausible to assume that the same holds true for bodily actions. We, therefore, think that various social cues (including bodily social interaction information) are integrated in a non-additive fashion during recognition to obtain a social percept of other persons.

The current study examined the effect of viewpoint on the visual recognition of three social interactions. Viewpoint influenced the visual recognition of the three social interactions in terms either of RT or d′. These observations extend previous knowledge about viewpoint-dependencies in the visual recognition of individual body postures (Reed et al., 2003) and biological motion of single persons (Troje et al., 2005) to two person interactions and show that not all social interactions are encoded in a view invariant manner. The correlation of the joint velocities with actual recognition performance indicates that joint velocities of the upper body, the arms, and the lower leg correlate well with recognition performance.

### Conflict of interest statement

The authors declare that the research was conducted in the absence of any commercial or financial relationships that could be construed as a potential conflict of interest.
